# Dasatinib reverses drug resistance by downregulating MDR1 and Survivin in Burkitt lymphoma cells

**DOI:** 10.1186/s12906-020-2879-8

**Published:** 2020-03-14

**Authors:** Mitsuki Tabata, Masanobu Tsubaki, Tomoya Takeda, Keisuke Tateishi, Katsumasa Tsurushima, Motohiro Imano, Takao Satou, Toshihiko Ishizaka, Shozo Nishida

**Affiliations:** 1 Division of Pharmacotherapy, Kindai University Faculty of Pharmacy, Kowakae, Higashi-Osaka, 577-8502 Japan; 2 Department of Phamacy, Sakai City Medical Center, Sakai, Japan; 3 Department of Surgery, Kindai University Faculty of Medicine, Osakasayama, Osaka Japan; 4 Department of Pathology, Kindai University Faculty of Medicine, Osakasayama, Osaka Japan

**Keywords:** Drug resistance, MDR1, Survivin, Src, Dasatinib

## Abstract

**Background:**

Current chemotherapies for Burkitt lymphoma (BL) have dramatically improved its clinical outcome. However, chemoresistance can lead to chemotherapy failure and very poor prognosis; thus, novel strategies are urgently required for patients with drug-resistant BL. To investigate the mechanisms underlying drug resistance in BL, we established drug-resistant BL cell lines: HS-Sultan/ADM (adriamycin-resistant), HS-Sultan/VCR (vincristine-resistant), HS-Sultan/DEX (dexamethasone-resistant), and HS-Sultan/L-PAM (melphalan-resistant).

**Methods:**

Drug transporter and survival factor expression were investigated the using western blotting and real time polymerase chain reaction. Cell survival was analyzed by trypan blue dye exclusion method.

**Results:**

The established cell lines acquired cross-resistance to adriamycin, vincristine, dexamethasone, and melphalan and exhibited 50% inhibitory concentration values 106-, 40-, 81-, and 45-fold higher than the parental cell lines, respectively. We found that protein and mRNA expression of MDR1 and Survivin were higher in drug-resistant BL cells than in the parent cells. Treatment with verapamil, an MDR1 inhibitor, or Survivin siRNA alongside each anti-cancer drug suppressed the proliferation of all drug-resistant BL cells. Src kinase activity was higher in all resistant cell lines than the parental cells; suppressing Src with dasatinib restored drug sensitivity by reducing MDR1 and Survivin expression.

**Conclusions:**

MDR1 and Survivin upregulation are responsible for resistance to conventional drugs and dasatinib can restore drug sensitivity by reducing MDR1 and Survivin expression in drug-resistant BL cells. Src inhibitors could therefore be a novel treatment strategy for patients with drug resistant BL.

## Background

Burkitt lymphoma (BL) is a fast-growing B-cell malignancy that accounts for 1–5% of acute lymphoblastic leukemias and non-Hodgkin lymphomas (NHL) [1]. Since BL is relatively sensitive to chemotherapy, the current high-dose/intensive chemotherapy and rituximab treatment achieves a three-year overall survival rate of over 80% [2–5]. However, chemoresistance can result in chemotherapy failure [6]. Patients with relapsed/refractory BL show a median overall survival of just 2.8 months [7]; thus, novel strategies are urgently required for patients with drug-resistant BL. ATP-binding cassette transporters, including multiple drug resistance 1 (MDR1, ABCB1), multidrug resistance-associated protein 1 (MRP1, ABCC1), and breast cancer resistance protein (BCRP, ABCG2) translocate drugs across the plasma membrane. The lung resistance protein 1 (LRP1) transports drugs away from their target molecules via cytoplasmic vesicles or pump molecules. The upregulation of these molecules has leads to cause drug resistance by reducing intracellular anti-cancer drug accumulation [8–12], thus is regarded as a marker of poor prognosis [13–18]. Apoptosis evasion, a hallmark of cancer, is responsible for both carcinogenesis and chemoresistance in various tumors. Bcl-2 and Bcl-XL, a member of anti-apoptotic Bcl-2 family proteins, suppress apoptosis by involving mitochondrial outer membrane permeability [19], yet inhibitor of apoptosis (IAP) family proteins do so by inhibiting caspase activity [20, 21]. Most anti-cancer drugs suppress tumor proliferation by inducing apoptosis, hence the overexpression of anti-apoptotic proteins results in drug resistance [22–25]. To understand drug resistance, further studies are required on the underlying intracellular signaling pathways.

Src kinase is a non-receptor tyrosine kinase which was involved in gene expression, immune responses, cell adhesion, cell cycle progression, apoptosis, migration, and transformation. Since it is responsible for tumor growth, metastasis, and angiogenesis, Src has been targeted for cancer treatment [26, 27]. In addition, Src is overactivated in various B lymphoma cell lines and patient-derived lymphoma, and inhibition of Src by PP1 and PP2 suppresses cell proliferation and induces cell death in BL cell line BAJB [28–30]. It has also been indicated that activation of Fyn and Syk by latent membrane protein 1 induces the Src/Akt pathway, which promotes cell proliferation and survival in Epstein–Barr virus-positive BL cells [31]. Our previous study showed that activation of Src induces multidrug resistance to anticancer drugs in multiple myeloma cells [32]. However, it is unclear whether activation of Src is involved with anticancer drug resistance in BL. Dasatinib is a therapeutic agent for chronic myeloid leukemia and a dual inhibitor of BCR/ABL and Src family kinases [33]. Src inhibition by dasatinib has been reported to resensitize drug-resistant cells to anti-cancer drugs [32, 34–36]; however, the effect of dasatinib in drug-resistant BL has not yet been investigated.

This study investigated the mechanisms of anticancer drug resistance and established therapeutic strategies for patients with drug-resistant BL.

## Methods

### Chemicals and reagents

Melphalan, adriamycin, vincristine, RPMI1640 medium, pepstatin, leupeptin, calpain inhibitor, phosphatase inhibitor cocktail I/II, and phenylmethylsulfonyl fluoride were purchased from Sigma (St. Louis, MO, USA). Dexamethasone, verapamil, 4-(2-hydroxyethyl)-1-piperazineethanesulfonic acid (HEPES), Tris-HCl (pH 7.4), EDTA, NP-40, sodium orthovanadate, and bicinchoninic acid protein-assay kit were purchased from Wako (Tokyo, Japan). Dasatinib was purchased from ChemieTek (Indianapolis, USA). Fetal bovine serum, penicillin, and streptomycin were purchased from Gibco (Carlsbad, CA, USA).

Melphalan, dexamethasone, and dasatinib were soluble in dimethyl sulfoxide, attenuated in phosphate-buffed saline (PBS); pH 7.4, and filtrated through 0.45 μm syringe (Iwaki Glass, Tokyo, Japan) filter before use. Adriamycin and vincristine were soluble in PBS. Verapamil was soluble in ultrapure water, and filtrated through 0.45 μm syringe (Iwaki Glass) filter before use.

### Cell culture

The human Burkitt lymphoma cell line HS-Sultan was provided from the DS Pharma Biomedical (EC87012701, Osaka, Japan). These cells were cultured in RPMI1640 medium (Sigma) including 100 μg/mL penicillin (Gibco), 10% fetal bovine serum (Gibco), 100 U/mL streptomycin (Gibco), and 25 mM HEPES (pH 7.4; Wako).

### Induction of anti-cancer drug resistance

HS-Sultan cells with acquired resistance to adriamycin, vincristine, dexamethasone, or melphalan were produced as previously described [12, 37, 38].

### Cell proliferation and survival assay

The effect of verapamil, adriamycin, vincristine, dexamethasone, melphalan, dasatinib, or Survivin siRNA on cell survival and proliferation was assessed using the trypan blue dye exclusion assay as previously described [12].

### Western blotting

Cytoplasmic cell fraction was collected by using cell lysis buffer (20 mM Tris-HCl (pH 8.0; Wako), 2 mM EDTA (Wako), 0.5% NP-40 (Wako), 1 μM pepstatin (Sigma), 1 μM leupeptin (Sigma), 2 mM sodium orthovanadate (Wako), 1 μM calpain inhibitor (Sigma), phosphatase inhibitor cocktail I/II (Sigma), and 1 mM phenylmethylsulfonyl fluoride (Sigma)). The protein contained amount of these fraction was evaluated using a bicinchoninic acid protein-assay kit (Wako). The extracts (40 μg of protein) were separated on sodium dodecyl sulfate polyacrylamide gels and transferred to polyvinyl difluoride membranes (GE Healthcare, Buckinghamshire, UK). The membranes were reacted with the following antibodies: anti-Bcl-2, anti-Bcl-xL, anti-Survivin, anti-MDR1, anti-BCRP, anti-MRP1, anti-LRP1 (Santa Cruz Biotechnologies, CA, USA), anti-phospho-Src (Tyr527), anti-Src (Cell Signaling Technology, Beverly, MA), and anti-β-actin (Sigma) as an internal control. The membranes were reacted with horseradish peroxidase-coupled secondary antibodies (GE Healthcare) for 1 h at room temperature and proteins were assessed using a Luminata Forte (Merck Millipore, Nottingham, UK).

### Quantitative real-time polymerase chain reaction (PCR)

The expression of MDR1 and Survivin mRNA was assessed using a Thermal Cycler Dice Real-Time system (Takara Biomedical) as previously described [12].

### RNA interference

Transfection of Survivin siRNA (siRNAs; HSS179403) were performed using LipofectAMINE™ 2000 reagent (Invitrogen) according to the manufacturer’s protocol as previously described [12].

### Statistical analysis

All data are demonstrated as the mean ± standard deviation of five independent experiments. All analysis were carried out by ANOVA with Dunnett’s test. *P* values of < 0.05 were regarded significant. Drug interactions were measured based on the combination index (CI), as previously described [24, 39].

## Results

### Drug sensitivity of established adriamycin-, vincristine-, dexamethasone-, and melphalan-resistant BL cell lines

**Fig. 1:**
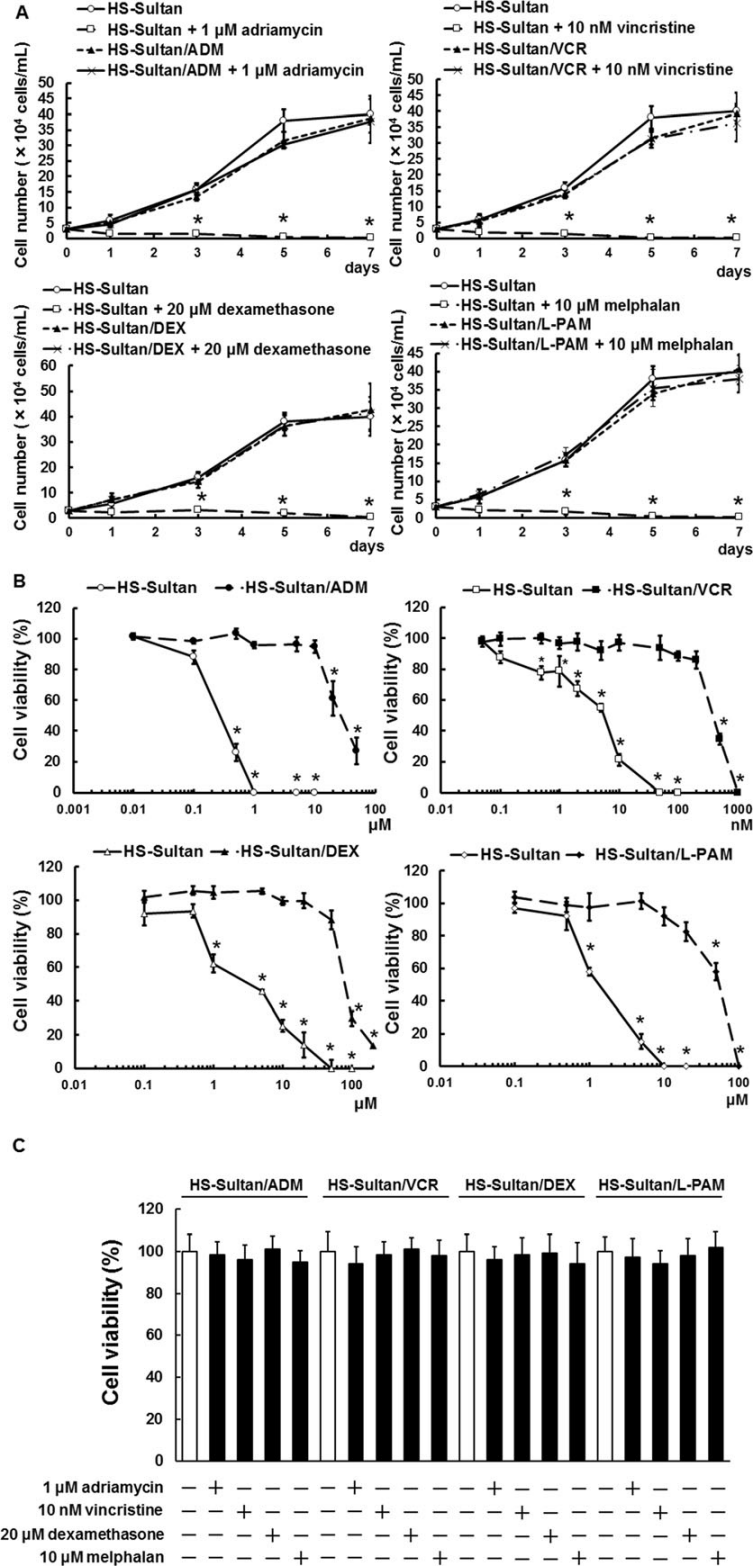
HS-Sultan/ADM, HS-Sultan /VCR, HS-Sultan /DEX, and HS-Sultan /L-PAM cell production and viability with various drugs. **a** HS-Sultan, HS-Sultan/ADM, HS-Sultan/VCR, HS-Sultan/DEX, and HS-Sultan/L-PAM cells were cultured with the represented concentrations of adriamycin, vincristine, dexamethasone, and melphalan. Cell number was appreciated using a trypan blue dye exclusion assay after 1, 3, 5, or 7 days. Results are notable example of five independent experiments. *p < 0.01 vs. untreated HS-Sultan cells (ANOVA with Dunnett’s test). **b** HS-Sultan, HS-Sultan/ADM, HS-Sultan/VCR, HS-Sultan/DEX, and HS-Sultan/L-PAM cells were cultured with the represented concentrations of adriamycin, vincristine, dexamethasone, and melphalan for 72 h. Cell viability was appreciated using a trypan blue dye exclusion assay. Results are notable example of five independent experiments. *p < 0.01 vs. control (ANOVA with Dunnett’s test). **c** Cross-resistance of drug-resistant BL cell lines. HS-Sultan/ADM, HS-Sultan/VCR, HS-Sultan/DEX, and HS-Sultan/L-PAM cells were cultured with 1 μM adriamycin, 10 nM vincristine, 20 μM dexamethasone, or 10 μM melphalan for 72 h. Cell number was detected using a trypan blue dye exclusion assay

We found that our established resistant cell lines, HS-Sultan/ADM (adriamycin-resistant), HS-Sultan/VCR (vincristine-resistant), HS-Sultan/DEX (dexamethasone-resistant), and HS-Sultan/L-PAM (melphalan-resistant), showed similar proliferation to the parental HS-Sultan cells; administration with adriamycin, vincristine, dexamethasone, and melphalan did not induced cell death in HS-Sultan/ADM, HS-Sultan/VCR, HS-Sultan/DEX, and HS-Sultan/L-PAM cells, but induced cell death in HS-Sultan cells (Fig. 1a). The IC50 values of the HS-Sultan cells for adriamycin, vincristine, dexamethasone, and melphalan were 0.221, 0.0073, 3.777, and 1.424 μM, respectively. In contrast, the IC50 values of HS-Sultan/ADM, HS-Sultan/VCR, HS-Sultan/DEX, and HS-Sultan/L-PAM cells for adriamycin, vincristine, dexamethasone, and melphalan were 23.471, 0.290, 304.919, and 64.558 μM, respectively, 106-, 40-, 81-, and 45-fold higher than those of the HS-Sultan cells (Fig. 1b). Furthermore, all resistant cell lines acquired cross-resistance to adriamycin, vincristine, dexamethasone, and melphalan (Fig. 1c).

### MDR1 and Survivin expression levels increased in drug-resistant BL cell lines

**Fig. 2:**
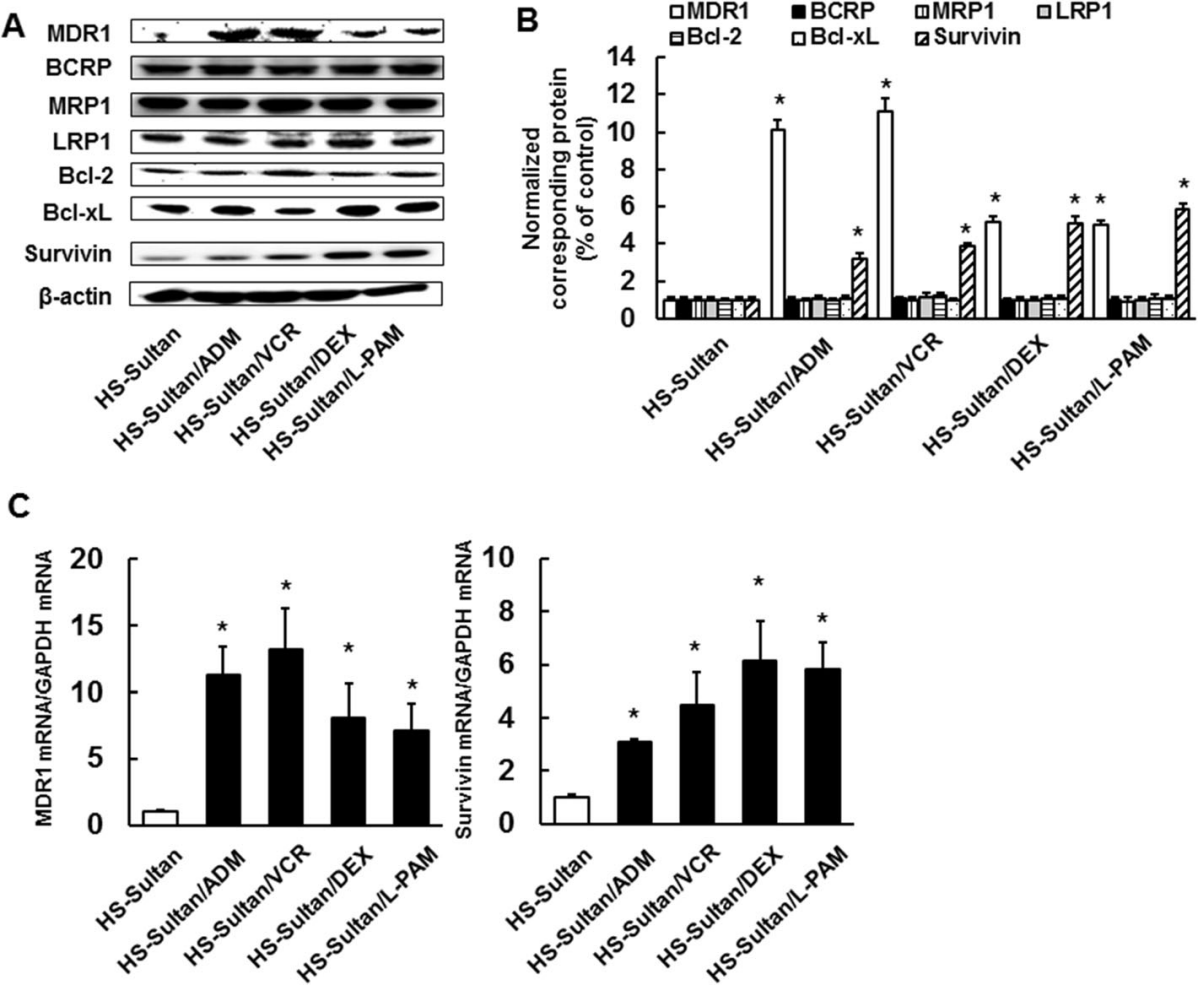
Expression levels of drug resistance-related proteins in HS-Sultan/ADM, HS-Sultan/VCR, HS-Sultan/DEX, and HS-Sultan/L-PAM cells. **a** Expression levels of a series of efflux pumps and anti-apoptosis proteins were assessed by western blotting analysis. Cytoplasmic cell fractions were extracted and performed to SDS-PAGE/immunoblotting with anti-MDR1, anti-BCRP, anti-MRP1, anti-LRP1, anti-Bcl-2, anti-Bcl-xL, and anti-Survivin antibodies. Anti-β-actin antibodies were used as an internal control. **b** Quantification of MDR1, BCRP, MRP1, LRP1, Bcl-2, BcL-xL, or Survivin levels, normalized to the amount of the β-actin. Results are notable example of five independent experiments. *p < 0.01 vs. control cells (ANOVA with Dunnett’s test). **c** mRNA expression of MDR1 and Survivin analyzed by real-time PCR. The results were normalized to GAPDH mRNA levels. Results are notable example of five independent experiments. *p < 0.01 vs. control (ANOVA with Dunnett’s test)

We investigated the expression levels of a series of efflux pumps and apoptosis-related proteins in HS-Sultan, HS-Sultan/ADM, HS-Sultan/VCR, HS-Sultan/DEX, and HS-Sultan/L-PAM cells. Expression of MDR1 and Survivin protein levels were substantially elevated in all resistant cells than the parental cells; however, Bcl-2, Bcl-xL, MRP1, LRP1, and BCRP expression did not change (Fig. 2a, b). Expression of MDR1 and Survivin mRNA levels were also elevated in all resistant cell lines than in the parental cells (Fig. 2c), suggesting that overexpressed expression of MDR1 and Survivin play an significant role in acquired drug resistance.

### Verapamil or Survivin siRNA treatment reversed adriamycin, vincristine, dexamethasone, and melphalan resistance

**Fig. 3:**
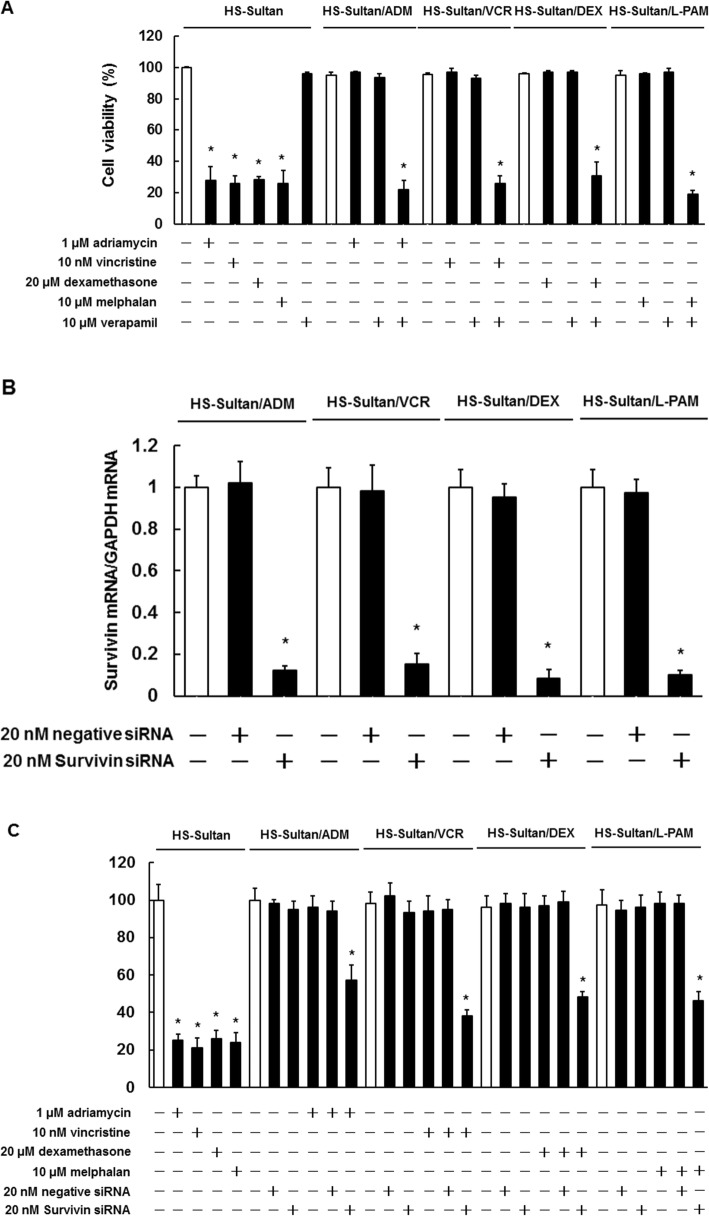
Effects of MDR1 and Survivin inhibitors on HS-Sultan/ADM, HS-Sultan/VCR, HS-Sultan/DEX, and HS-Sultan/L-PAM cell drug sensitivity. **a** HS-Sultan, HS-Sultan/ADM, HS-Sultan/VCR, HS-Sultan/DEX, and HS-Sultan/L-PAM cells were incubated with the represented concentrations of adriamycin, vincristine, dexamethasone, melphalan, and verapamil for 72 h. Detection of dead cells number was performed by trypan blue staining. Results are notable example of five independent experiments. *p < 0.01 vs. control (ANOVA with Dunnett’s test). **b** mRNA expression of Survivin analyzed by real-time PCR. The results were normalized to GAPDH mRNA levels and are notable example of five independent experiments. *p < 0.01 vs. control (ANOVA with Dunnett’s test). **c** HS-Sultan, HS-Sultan/ADM, HS-Sultan/VCR, HS-Sultan/DEX, and HS-Sultan/L-PAM cells were incubated with the represented concentrations of adriamycin, vincristine, dexamethasone, melphalan, and Survivin siRNA for 72 h. Detection of dead cells number was performed by trypan blue staining. Results are notable example of five independent experiments. *p < 0.01 vs. control cells (ANOVA with Dunnett’s test)

To determine whether MDR1 and Survivin were involved in acquired drug resistance, we assessed the viability of HS-Sultan/ADM, HS-Sultan/VCR, HS-Sultan/DEX, and HS-Sultan/L-PAM cells treated with verapamil, an MDR1 inhibitor, or Survivin siRNA. As shown in Fig. 3a, the combined treatment of verapamil with adriamycin, vincristine, dexamethasone, or melphalan induced cell death in all resistant cells. Verapamil treatment alone did not affect the viability of either the drug-sensitive or drug-resistant cells. The Survivin siRNA treatment (20 nM) was sufficient to suppress Survivin expression (Fig. 3b) and the viability of all resistant cell lines when combined with each anti-cancer agent (Fig. 3c). These results suggest that targeting MDR1 and Survivin could overcome drug resistance.

### Dasatinib overcame drug resistance by downregulating MDR1 and Survivin

**Fig. 4:**
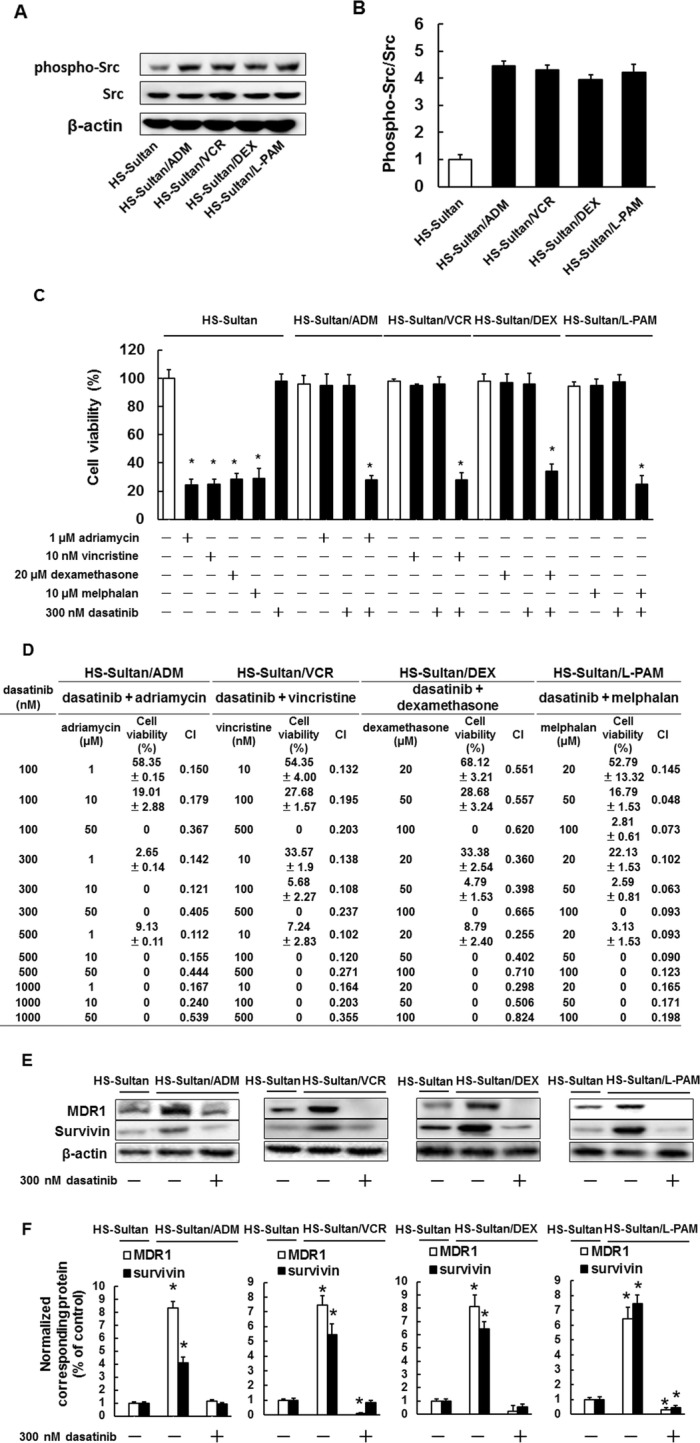
The src inhibitor dasatinib reversed drug resistance in HS-Sultan/ADM, HS-Sultan/VCR, HS-Sultan/DEX, and HS-Sultan/L-PAM cells. **a** Phosphorylated Src expression levels were assessed by western blotting analysis. Cytoplasmic cell fractions were extracted and performed to SDS-PAGE/immunoblotting with anti-Src antibodies. Anti-β-actin antibodies were used as an internal standard. **b** Quantification of phosphorylated Src levels. Results were corrected according to total Src levels and are notable example of five independent experiments. *p < 0.01 vs. control cells (ANOVA with Dunnett’s test). **c** HS-Sultan, HS-Sultan/ADM, HS-Sultan/VCR, HS-Sultan/DEX, and HS-Sultan/L-PAM cells were incubated with the represented concentrations of adriamycin, vincristine, dexamethasone, melphalan, and dasatinib for 72 h. Detection of dead cells number was performed by trypan blue staining. Results are notable example of five independent experiments. *p < 0.01 vs. control cells (ANOVA with Dunnett’s test). **d** Combination index (CI) values for combination treatment of dasatinib and adriamycin, vincristine, dexamethasone, or melphalan were calculated. CI values less than 1.0 indicate synergy, while CI values greater than 1 indicate antagonism. **e** MDR1 and Survivin expression levels were assessed by western blotting analysis. HS-Sultan, HS-Sultan/ADM, HS-Sultan/VCR, HS-Sultan/DEX, and HS-Sultan/L-PAM cells were incubated with 300 nM dasatinib for 72 h. Cytoplasmic cell fractions were extracted and performed to SDS-PAGE/immunoblotting with anti-MDR1 and anti-Survivin antibodies. Anti-β-actin antibodies were used as an internal standard. **f** Quantification of MDR1 and Survivin levels, normalized to the amount of the β-actin. Results are notable example of five independent experiments. *p < 0.01 vs. control cells (ANOVA with Dunnett’s test)

We investigated whether Src affected drug resistance in HS-Sultan/ADM, HS-Sultan/VCR, HS-Sultan/DEX, and HS-Sultan/L-PAM cells. Src phosphorylation levels were higher in all resistant BL cells than in HS-Sultan cells (Fig. 4a, b) and dasatinib restored drug sensitivity in all resistant cells at the indicated concentration (Fig. 4c). In addition, the interactions among dasatinib and adriamycin, vincristine, dexamethasone, or melphalan were analyzed using the Chou–Talalay method. Per the combination drug concentrations shown in Fig. 4d, the CI ranged from 0.824 to 0.049, indicating the synergistic effect of these combinations (Fig. 4d). Next, we investigated MDR1 and Survivin expression levels in resistant cells treated with dasatinib. Dasatinib treatment reduced MDR1 and Survivin expression levels to the same extent in all resistant cells compared to the parental HS-Sultan cells (Fig. 4e, f), indicating that Src inhibition could restore drug sensitivity by reducing overexpression of MDR1 and Survivin in acquired drug-resistant BL cells.

## Discussion

To investigate the mechanisms of anti-cancer drug resistance, we established drug-resistant BL cell lines, including HS-Sultan/ADM, HS-Sultan/VCR, HS-Sultan/DEX, and HS-Sultan/L-PAM cells. The resistant cells showed similar growth to parental HS-Sultan cells and displayed higher IC50 values than the parental cells for adriamycin, vincristine, dexamethasone, and melphalan, respectively. In addition, all resistant cells acquired cross-resistance to other anti-cancer drugs.

In this study, MDR1 and Survivin protein and mRNA expression levels were elevated in all resistant cells than in the parental cells. Moreover, verapamil and Survivin siRNA reversed adriamycin, vincristine, dexamethasone, and melphalan resistance. It has been reported that MDR1 overexpression in Namalwa cells, a human BL cell line, promotes the efflux of adriamycin and vincristine and induces drug resistance [40]. It has also been indicated that inhibition of MDR1/P-glycoprotein function by verapamil re-sensitizes the cytotoxic effect of vincristine in vincristine-resistant Namalwa and Raji cells [41]. In addition, MDR1/P-glycoprotein overexpression induces resistance to treatment with the CHOP (cyclophosphamide, adriamycin, vincristine, prednisolone) regimen in patients with NHL including BL [42]. The expression of Survivin, a member of the IAP protein family that inhibits caspase activity, is higher in patients with BL than in patients with reactive lymphoid hyperplasia [43]. In addition, high expression of Survivin in patients with BL was associated with resistance to chemotherapy compared to low expression of Survivin in patients with BL [44]. It has been reported that YM155, a Survivin inhibitor, suppresses tumor growth and prolongs the survival time in SCID mice bearing the Ramos BL cell line compared to rituximab [45]. These findings suggest that MDR1 and Survivin are correlated with drug resistance, and inhibition of these factors re-sensitize the anticancer drugs.

Src kinase is responsible for tumor survival, hence this molecule has been reported as an attractive target for cancer treatment [26, 27, 46]. Dasatinib, an Src inhibitor, has been reported to resensitize drug-resistant cells to anti-cancer drugs [32, 34–36, 47]. In this study, Src activity was elevated in all resistant cells than in the parental cells, while dasatinib, an Src inhibitor, reversed adriamycin, vincristine, dexamethasone, and melphalan resistance. In addition, dasatinib reduced Src phosphorylation and MDR1 and Survivin protein expression in resistant cells to less than or equal to that of the parental cells. It has been indicated that dasatinib suppresses diffuse large B-cell lymphoma cell proliferation in vitro and tumor growth in vivo through inhibition of Src phosphorylation [29]. In addition, Src activation by gap junction beta-4-induced chemoresistance to gemcitabine and etoposide, in addition to dasatinib enhances the cytotoxic effect of gemcitabine in lung cancer [48]. It has been reported that dasatinib enhances the inhibitory effect of tumor cell growth by trametinib, a mitogen-activated protein kinase kinase inhibitor, in vitro and in vivo in various KRAS-mutant cancer cells, including lung, breast, colon, and pancreatic cancer cells [49]. Dasatinib has also been shown to increase cisplatin sensitivity in esophageal squamous cell carcinoma and adriamycin sensitivity in breast cancer by downregulating MDR1 [50, 51]. Moreover, dasatinib has been shown to reduce MDR1 and Survivin levels; increase Bim levels; and restore adriamycin, vincristine, dexamethasone, and melphalan sensitivity in drug-resistant multiple myeloma cells [32]. Our results clearly show the first evidence of an anticancer drug-resistant mechanism through the activation of Src in BL cells. In addition, dasatinib overcomes anticancer drug resistance via inhibition of Src phosphorylation and MDR1 and Survivin expression in BL cells and similarly drug-resistant multiple myeloma cells. Collectively, these findings suggest that dasatinib is re-sensitized to anticancer drugs in drug-resistant BL.

## Conclusions

In conclusion, we found that MDR1 and Survivin upregulation is responsible for resistance to conventional drugs. Moreover, dasatinib restores drug sensitivity by reducing MDR1 and Survivin levels in drug-resistant BL cells. Our findings indicate that Src inhibitors could be a novel strategy for treating patients with drug resistant BL.

## Supplementary information

**Additional file 1.**

**Additional file 2.**

**Additional file 3.**

## Data Availability

The analyzed data sets generated during the study are available from the corresponding author on reasonable request.

## Abbreviations

BCRP: Breast cancer resistance protein
BL: Burkitt lymphoma
IAP: Inhibitor of apoptosis
IC50: 50% inhibition concentration
LRP1: Lung resistance protein 1
MDR1: Multiple drug resistance 1
MRP1: Multidrug resistance-associated protein 1
NHL: Non-Hodgkin lymphomas
