# Supplementary figures and images for: Dasatinib reverses drug resistance by downregulating MDR1 and Survivin in Burkitt lymphoma cells
Source: BMC Complement Med Ther. 2020 Mar 14;20:84. doi: 10.1186/s12906-020-2879-8 (PMC7076888; doi:10.1186/s12906-020-2879-8)

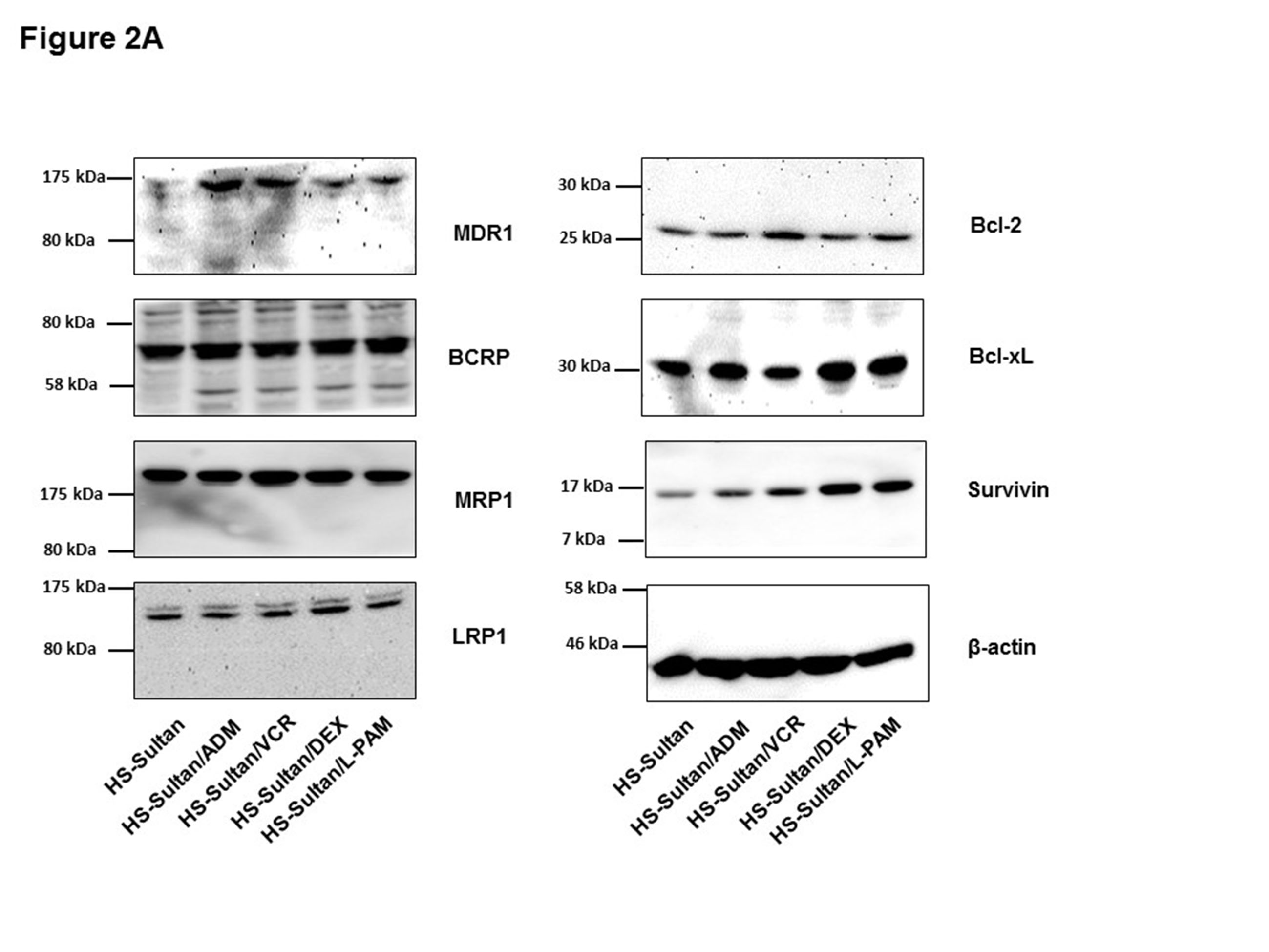

Supplement: Supplementary file 1 — Additional file 1. [file 12906_2020_2879_MOESM1_ESM.tif]

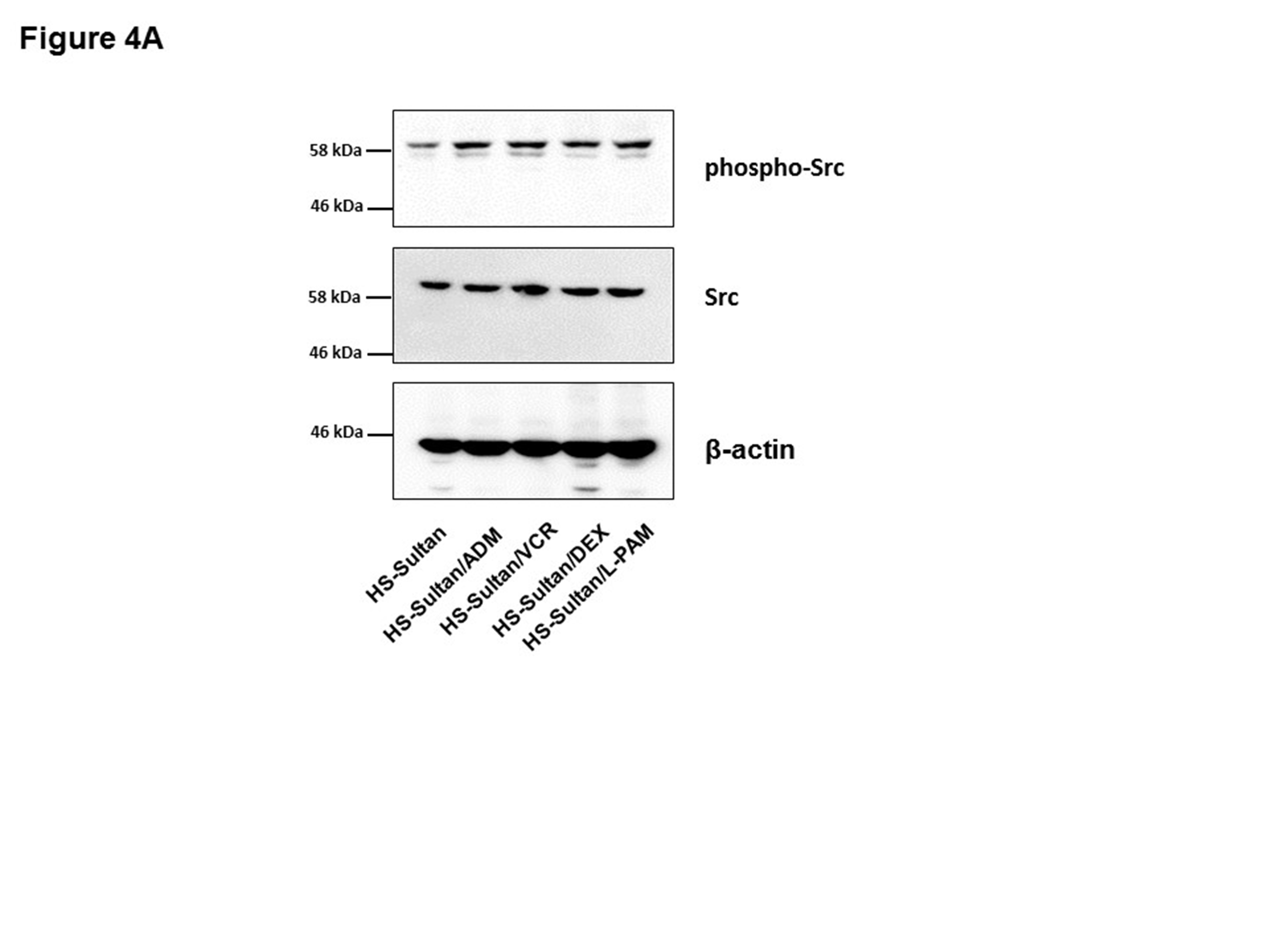

Supplement: Supplementary file 2 — Additional file 2. [file 12906_2020_2879_MOESM2_ESM.tif]

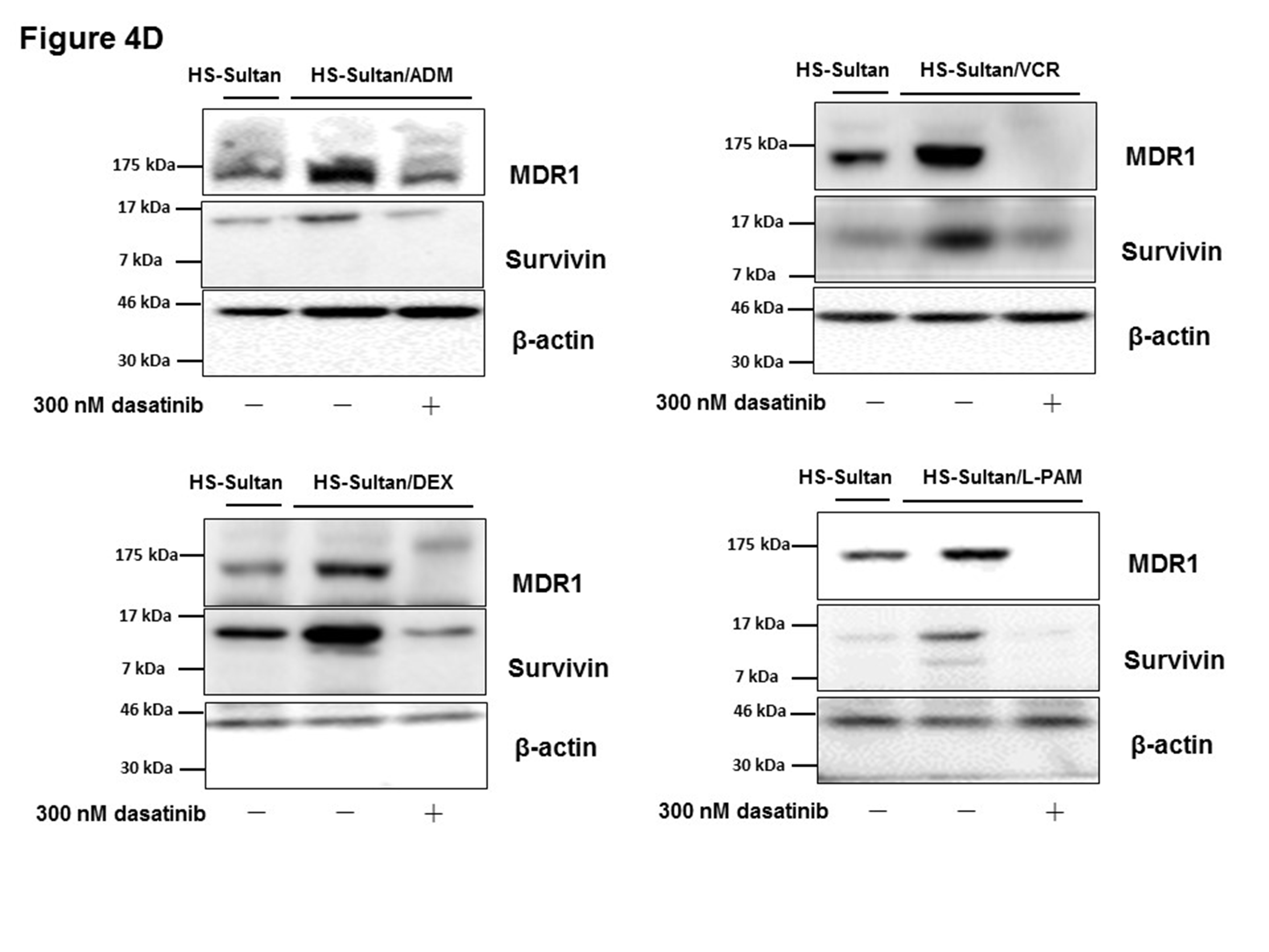

Supplement: Supplementary file 3 — Additional file 3. [file 12906_2020_2879_MOESM3_ESM.tif]
